
JOURNAL INFORMATION
==============================
NLM Title Abbreviation: ScientificWorldJournal
Journal ID: ScientificWorldJournal
NLM Journal ID: 101131163
Journal ID: TSWJ

The Scientific World Journal

ISSN: 2356-6140
EISSN: 1537-744X
Publisher: Wiley

ARTICLE INFORMATION
==============================
PMCID: PMC6084513
PMID: 30147561
DOI: 10.1100/tsw.2001.197
Article ID: 595026
Article version: 1
Subjects: Short Report

Control of Cell Survival and Proliferation in the Oligodendrocyte Cell Lineage

Raff Martin *
Tokumoto Yasu
Tang Dean
MRC Laboratory for Molecular Cell Biology, University College London, London WC1E 6BT, UK
*Martin Raff: m.raff@ucl.ac.uk
Publication date: 2001
Electronic publication date: 2001 Apr 4
Volume: 1
First page: 90
Last page: 90
Copyright: Copyright © 2001 Martin Raff et al.
Copyright year: 2001
License: This is an open access article distributed under the Creative Commons Attribution License, which permits unrestricted use, distribution, and reproduction in any medium, provided the original work is properly cited.
License URL: https://creativecommons.org/licenses/by/3.0/

==============================
Miami *Nature Biotechnology* Short Reports
*TheScientificWorld* (2001) 1 (S3), 90SR
ISSN 1532-2246; DOI 10.1100/tsw.2001.197

### CONTROL OF CELL SURVIVAL AND PROLIFERATION IN THE OLIGODENDROCYTE CELL LINEAGE

Martin Raff*, Yasu Tokumoto and Dean Tang

MRC Laboratory for Molecular Cell Biology, University College London, London WC1E 6BT, UK
* m.raff@ucl.ac.uk

**INTRODUCTION.** The behavior of animal cells depends on both intracellular programs and extracellular signals that regulate these programs. Here we consider two types of cell behavior—cell survival and cell proliferation—in the oligodendrocyte cell lineage in the rodent optic nerve, one of the simplest parts of the central nervous system (CNS). Oligodendrocytes make myelin in the CNS. They develop from precursor cells (OPCs) that migrate into the developing optic nerve from the brain. The OPCs divide a limited number of times before they stop and terminally differentiate (1). The final number of oligodendrocytes in the optic nerve depends on how many OPCs migrate into the nerve, how many times each OPC divides before it terminally differentiates, and how much cell death occurs in the cell lineage (2). We begin by considering the control of cell survival and death and then consider the controls on OPC proliferation.

**CONTROL OF OLIGODENDROCYTE SURVIVAL.** Most animal cells require signals from other cells to survive (3), and oligodendrocytes are no exception (4). They are overproduced and then culled by apoptosis in a competition for limiting amounts of survival signals provided by their neighbors, especially by the axons of retinal ganglion cells that pass through the optic nerve on their way to the brain (2). Newly formed oligodendrocytes require signals from axons in order to survive: about half of those generated manage to contact an axon and live, while the rest fail to do so and die (2). This strategy serves to match the number of oligodendrocytes to the number and length of axons requiring myelination. One of the survival signals provided by the axons is the neuregulin glial growth factor (GGF): experimentally increasing GGF in the developing optic nerve decreases the normal death of oligodendrocytes, while neutralizing GGF increases this death (5).

**CONTROL OF OPC PROLIFERATION.** Each OPC has a built-in timer that helps determine when the cell stops dividing and differentiates (6). The timer depends on various intracellular proteins that either increase or decrease over time as the cell proliferates (6,7). Although the timer is cell-intrinsic, it depends on extracellular signals for its normal operation—including PDGF, which stimulates OPC proliferation (8), and thyroid hormone (TH), which helps trigger cell-cycle withdrawal and differentiation (9). If purified OPCs from the developing rat optic nerve are cultured in the absence of PDGF, they immediately stop dividing and differentiate; if they are cultured in the presence of PDGF and TH, they tend to differentiate on a similar schedule to that seen *in vivo* (1); if TH is omitted from the culture medium, most of the cells continue to proliferate and do not differentiate (9).

Surprisingly, OPCs cultured in PDGF without TH can be cultured indefinitely (11). They maintain telomerase activity and seem not to undergo crisis or replicative cell senescence. Cells that have been cultured for more than 1½ years seem not to have acquired immortalizing mutations, as they maintain p53-dependent and Rb-dependent checkpoints (12). Although cell-cycle inhibitors, including INK and CIP/KIP family cyclin-dependent kinase (Cdk) inhibitors and p53, increase as the cells proliferate, the cells do not withdraw from the cycle, perhaps because some Cdk's and G1 cyclins increase as well (12). This finding suggests that rodent somatic cells do not have a built-in cell-division counter that limits their proliferation in the way that human cells do, although they do have cell-cycle checkpoints that can arrest the cell cycle if the cells are stimulated to proliferate in an uncontrolled way.

**REFERENCES.**

1. Temple, S. and Raff, M.C. (1986) Cell 44, 773–779
2. Barres, B.A. and Raff, M.C. (1999) J. Cell Biol. 147, 1123–1128
3. Raff, M.C. (1992) Nature 356, 397–400
4. Barres, B.A., Hart, I.K., Coles, H.S.R., Burne, J.F., Voyvodic, J.T., Richardson, W.D., Fernandez, P.A., Tang, D., Cheng, L., Prochiantz, A., Mudge, A., and Raff, M. (2000) Neuron 28, 81–90
5. Durand, B. and Raff, M. (2000) Bioessays 22, 64–71
6. Kondo, T. and Raff, M. (2000) EMBO J. 19, 1998–2007
7. Raff, M.C., Lillien, L.E., Richardson, W.D., Burne, J.F., and Noble, M.D. (1988) Nature 333, 562–565
9. Barres, B.A., Lazar, M.A., and Raff, M.C. (1994). Development 120, 1097–1108
10. Gao, F.B., Apperly, J., and Raff, M.C. (1998) Dev. Biol. 197, 54–66
11. Tang, D.G., Tokumoto, Y.M., and Raff, M.C. (2000) J. Cell Biol. 148, 971–984
12. Tang, D.G., Tokumoto, Y.M., Apperly, J., Lloyd, A., and Raff, M.C. (2000) Submitted
